# Targeting EMP3 suppresses proliferation and invasion of hepatocellular carcinoma cells through inactivation of PI3K/Akt pathway

**DOI:** 10.18632/oncotarget.5414

**Published:** 2015-10-12

**Authors:** Yi-Hsien Hsieh, Shu-Ching Hsieh, Chien-Hsing Lee, Shun-Fa Yang, Chun-Wen Cheng, Meng-Ju Tang, Chia-Liang Lin, Chu-Liang Lin, Ruey-Hwang Chou

**Affiliations:** ^1^ Department of Biochemistry, School of Medicine, Chung Shan Medical University, Taichung, Taiwan; ^2^ Clinical Laboratory, Chung Shan Medical University Hospital, Taichung, Taiwan; ^3^ Institute of Medicine, Chung Shan Medical University, Taichung, Taiwan; ^4^ Graduate Institute of Medical Sciences, Chang Jung Christian University, Tainan, Taiwan; ^5^ Division of Pediatric Surgery, Department of Surgery, Children's Hospital of China Medical University, Taichung, Taiwan; ^6^ Institute of Biochemistry, Microbiology and Immunology, Chung Shan Medical University, Taichung, Taiwan; ^7^ Graduate Institute of Cancer Biology and Center for Molecular Medicine, China Medical University, Taichung, Taiwan; ^8^ Department of Biotechnology, Asia University, Taichung, Taiwan

**Keywords:** epithelial membrane protein-3, proliferation, migration, invasion, hepatocellular carcinoma

## Abstract

Epithelial membrane protein-3 (EMP3), a typical member of the epithelial membrane protein (EMP) family, is epigenetically silenced in some cancer types, and has been proposed to be a tumor suppressor gene. However, its effects on tumor suppression are controversial and its roles in development and malignancy of hepatocellular carcinoma (HCC) remain unclear. In the present study, we found that EMP3 was highly expressed in the tumorous tissues comparing to the matched normal tissues, and negatively correlated with differentiated degree of HCC patients. Knockdown of EMP3 significantly reduced cell proliferation, arrested cell cycle at G1 phase, and inhibited the motility and invasiveness in accordance with the decreased expression and activity of urokinase plasminogen activator (uPA) and matrix metalloproteinase 9 (MMP-9) in HCC cells. The *in vivo* tumor growth of HCC was effectively suppressed by knockdown of EMP3 in a xenograft mouse model. The EMP3 knockdown-reduced cell proliferation and invasion were attenuated by inhibition of phosphatidylinositol 3-kinase (PI3K) or knockdown of Akt, and rescued by overexpression of Akt in HCC cells. Clinical positive correlations of EMP3 with p85 regulatory subunit of PI3K, p-Akt, uPA, as well as MMP-9 were observed in the tissue sections from HCC patients. Here, we elucidated the tumor progressive effects of EMP3 through PI3K/Akt pathway and uPA/MMP-9 cascade in HCC cells. The findings provided a new insight into EMP3, which might be a potential molecular target for diagnosis and treatment of HCC.

## INTRODUCTION

Hepatocellular carcinoma (HCC) is one of the most common cancers and a leading cause of cancer mortality worldwide, particularly in Southeast Asia including China, Taiwan, Korea and Japan, and sub-Saharan Africa [1, 2]. Development of HCC is a multistep process, in which the chronic infections of hepatitis B virus (HBV) and hepatitis C virus (HCV), and liver cirrhosis are closely relevant [2]. The disease is often diagnosed at an advanced stage due to the asymptomatic lesion in an early stage of HCC. Only around 30% of patients are eligible for curative therapies, such as surgery or ablation. Treatment options of the other intermediate/advanced metastasized HCC are limited as transcatheter arterial chemoembolization (TACE), radioembolization, and systemic therapy, such as chemotherapy and sorafenib, a multikinase inhibitor [3]. However, clinical outcomes of these unresectable HCC patients are still unfavorable. Therefore, understanding the molecular mechanisms of malignancy of HCC will provide novel molecular target for a potential therapeutic strategy.

In addition to late diagnosis of HCC patients, cancer metastasis is one of the critical causes of poor clinical outcome or mortality [4]. The process of metastatic spread of cancers is complicated including cell adhesion, migration, invasion, and proteolytic degradation of extracellular matrix (ECM) [5]. Urokinase plasminogen activator (uPA) [6] and matrix metalloproteinases (MMPs) [7] are involved in degradation of ECM, which is a critical step in the metastatic process. The uPA network consists of uPA, uPA receptor (uPAR), plasminogen, plasmin, and the negative regulators, plasminogen activator inhibitors (PAIs), PAI-1 and PAI-2 [6]. uPA activates a serine proteinase plasmin to degrade the components of ECM by proteolytic cleavage of inactive zymogen plasminogen, leading to promotion of invasion of cancer cells. Increase of uPA cascade [8] and activity of MMPs [9] have been demonstrated in invasion and metastasis of HCC. Thus, we aimed to investigate the upstream signaling of uPA and MMPs in malignancy of HCC in the current study.

Human epithelial membrane protein-3 (EMP3) located on chromosome 19q13.3 was first identified by homology screening of databases [10, 11]. EMP3 encodes a 163 amino-acid protein containing 4 transmembrane domains, and belongs to the peripheral myelin protein 22-kDa (PMP22) gene family of small hydrophobic membrane glycoproteins due to the high amino acid sequence homology [12]. The expression of EMP-3 is found in most tissues including liver, and higher in peripheral blood leukocytes, ovary, intestine and various embryonic tissues [11]. The functions of EMP3 are still unclear and controversial. EMP3 has been proposed to be a tumor suppressor gene, which is silenced by hypermethylation on its promoter region in glioma [13–16], neuroblastoma [13, 17], and non-small cell lung cancer [18]. Its promoter methylation concomitant with the loss of heterozygosity of chromosome 19q has been observed in the grade II oligodendroglioma patients at a higher risk of relapse and disease progression-free interval [19]. The potential tumor suppressive function of EMP3 has also been reported to be repressed in esophageal squamous cell carcinoma cell lines [20]. In contrast, other reports do not support EMP3 as a candidate of tumor suppressor gene and suggest its function in tumor aggressiveness. Overexpression of EMP3 is found in oligodendroglial tumors [21], and primary breast carcinomas without altering DNA methylation [22]. Recently, EMP3 has been demonstrated to promote cell proliferation and migration and its co-expression with ErbB2 is unfavorable for progression-free and metastasis-free survival in patients with upper urinary tract urothelial carcinoma [23].

Although EMP3 transcripts can be detected in liver, the roles of EMP3 in HCC development and malignancy remain unknown so far. In the present study, we demonstrated the potential roles of EMP3 in tumor aggressiveness of HCC cells, and found that EMP3 was highly expressed in the tumorous tissues comparing to non-tumorous tissues of HCC patients. Knockdown of EMP3 reduced cell proliferation, arrested cell cycle at G1 phase, and inhibited the abilities of cell migration and invasion accompanied with a decrease of the expression and activity of MMP-9 and uPA in HCC cells. The suppression of cell proliferation and malignancy caused by silencing EMP3 of HCC cells was mediated with down-regulation of phosphatidylinositol 3-kinase (PI3K)/Akt pathway. The study provided a new insight into EMP3, suggesting that EMP3 enhances malignancy of HCC cells, and it might be a potential molecular target for diagnosis and treatment of HCC patients.

## RESULTS

### EMP3 is highly expressed in tissue sections from HCC patients and in HCC cell lines

**Figure 1:**
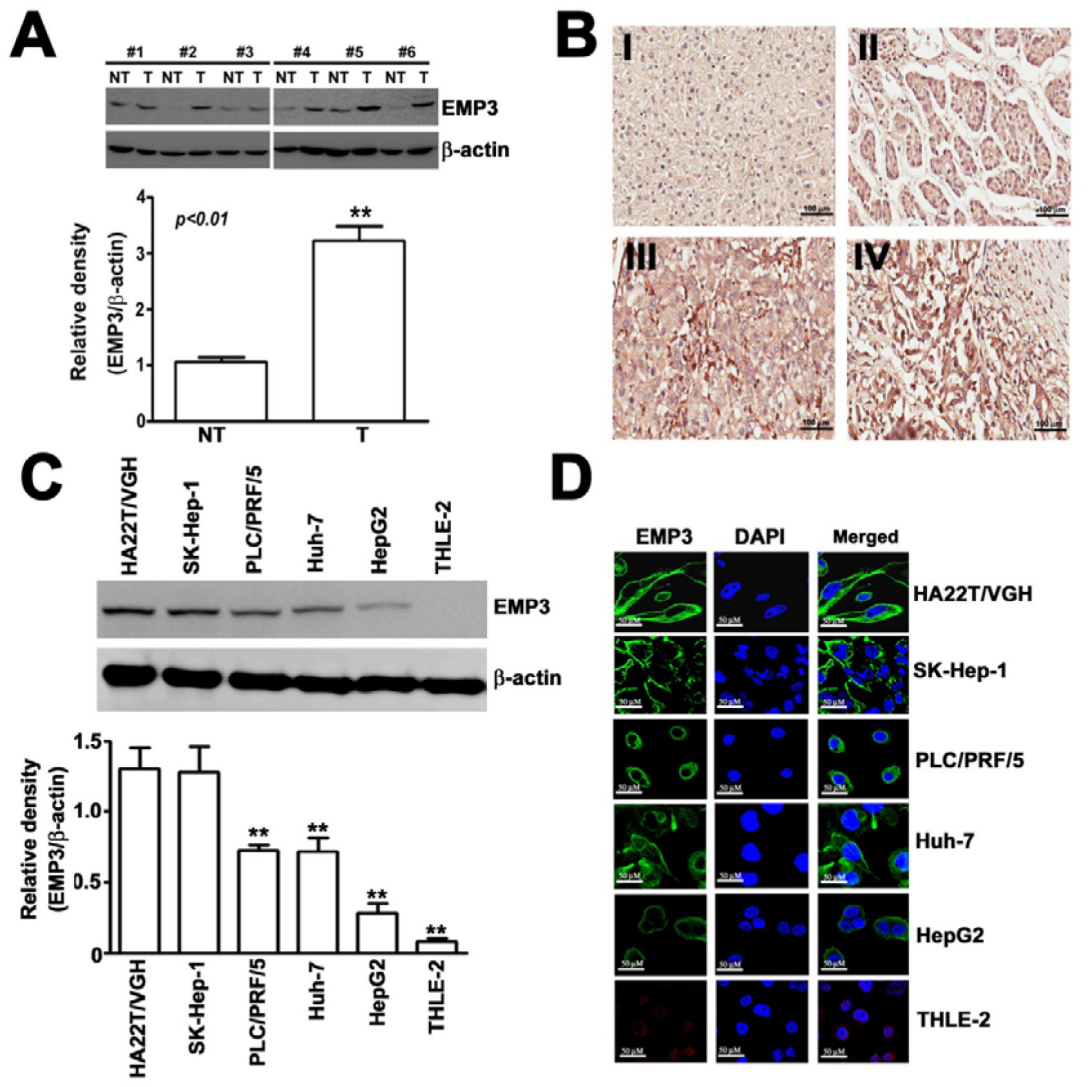
EMP3 is highly expressed in tissue sections from HCC patients and in HCC cell lines **A.** The expression of EMP3 was examined by immunoblotting. Upper panel: the representative results of the amounts of EMP3 in paired non-tumorous (NT) and tumorous (T) tissue samples from clinical HCC patients. Lower plot: the relative amounts of EMP3 normalized by β-actin from 16 NT/T paired HCC tissues. **, *P* < 0.01, compared with that of the non-tumorous (NT) tissues. **B.** The representative IHC staining of EMP3 from normal tissues (I) and different differentiated HCC tumorous (II-IV). Scale bars = 100 μm. **C.** The protein expression levels of EMP3 in different differentiated HCC cell lines, including poor differentiated HA22T/VGH and SK-Hep-1, and moderate differentiated PLC/PRF/5 and Huh-7, well differentiated HepG2 cells, and normal hepatic THLE-2 cells. The bottom plot was the quantitative amounts of EMP3 normalized by β-actin in each cell line from three independent experiments. **D.** The IF staining of EMP3 (green color) and DAPI staining of nucleus (blue color) in each cell line. Scale bars = 50 μm. Data are presented as the mean ± SE of at least three independent experiments. **, *p* < 0.01.

To clarify the correlation between EMP3 and development of human HCC, We examined the expression of EMP3 protein by Western blot in HCC tissues and matched non-tumorous tissues. Of 16 HCC tumors, 13 expressed higher levels of EMP3 than non-tumorous tissues (Figure 1A). Immunohistochemical staining was conducted on a tissue microarray containing samples from 74 patients of HCC tissues and 20 normal hepatic tissues. Higher levels of EMP3 in HCC tissues were evidenced compared with normal hepatic tissues (Figure 1B). Consistently, the expression of EMP3 was significantly negatively correlated with degree of differentiation in HCC patients (*p* < 0.031). In contrast, there was no significant correlation between EMP3 expressions in age, sex, and tumor stage of HCC tissues (Table 1). Furthermore, we confirmed expression of EMP3 in 5 human HCC cells (HA22T/VGH, SK-Hep-1, Huh-7, PLC/PRF/5 and HepG2) and one normal hepatic cell (THLE-2), the expression levels of EMP3 in poor differentiated HCC cell lines, HA22T/VGH and SK-Hep-1, were much higher than that in moderate differentiated PLC/PRF/5 and Huh-7 and well differentiated HepG2 cell lines, and lowest in THLE-2 normal hepatic cell line, as determined by immunoblotting (Figure 1C) and immunofluorescence (IF) staining (Figure 1D). Taken together, EMP3 was conversely associated with differentiation of HCC, suggesting its potential roles in malignancy of HCC.

**Table 1:** Expression of EMP3, p85, p-Akt, uPA, MMP-9 in relation to patient characteristics and pathological features of hepatocellular carcinoma

Characteristic	Number of patients (%)		*p Value*	Number of patients (%)		*p Value*	Number of patients (%)		*p Value*	Number of patients (%)		*p Value*	Number of patients (%)		*p Value*
	EMP3(-)	EMP3(+)		p85(-)	p85 (+)		p-Akt(-)	p-Akt(+)		uPA(-)	uPA(+)		MMP-9(-)	MMP-9(+)	
Total number of patients	35 (47.3)	39 (52.7)		19 (25.7)	55 (74.3)		36 (48.6)	38 (41.4)		18 (24.3)	56 (75.7)		12 (16.2)	62 (83.8)	
**Age (year)**															
<50	15 (46.9)	17(53.1)	0.568	9 (28.1)	23(71.9)	0.437	15(46.9)	17(53.1)	0.487	11(34.4)	21(65.6)	0.103	8 (25.0)	24 (75.0)	0.111
≥50	20 (47.6)	22 (52.4)		10 (23.8)	32 (76.2)		21 (50.0)	21 (50.0)		7(16.7)	35 (83.3)		4 (9.5)	38 (90.5)	
**Gender**															
Male	35 (48.6)	37(51.4)	0.274	19 (26.4)	53 (73.6)	0.551	34 (47.2)	38 (52.8)	0.233	18 (25)	54 (75)	0.570	12(16.7)	60 (83.3)	0.700
Female	0 (0)	2 (100)		0 (0)	2 (100)		2 (100)	0 (0)		0 (0)	2 (100)		0 (0)	2 (100)	
**Differentiated**															
well	5 (83.3)	1 (16.7)	**<0.031**	3 (50)	3 (50)	**<0.018**	4 (66.7)	2 (33.3)	**<0.037**	2 (33.3)	4 (66.7)	**<0.034**	2 (33.3)	4 (66.7)	**<0.009**
moderately	18 (56.3)	14 (43.7)		11 (34.4)	21(65.6)		20(62.5)	12 (37.5)		11 (34.4)	21 (65.6)		9 (28.1)	23(71.9)	
poorly	12 (33.3)	24 (66.7)		5 (13.9)	31 (86.1)		12 (33.3)	24 (66.7)		4 (11.1)	32 (88.9)		1 (2.8)	35 (97.2)	
**Tumor stage**															
I+II	18 (52.9)	16 (47.1)	0.254	4 (11.8)	30 (88.2)	**<0.016**	13 (38.2)	21 (61.8)	0.078	5 (14.7)	29 (85.3)	0.167	4 (11.8)	30 (88.2)	0.263
III+IV	17 (42.5)	23 (57.5)		15 (37.5)	25 (62.5)		23 (57.5)	27 (42.5)		13 (32.5)	27 (67.5)		8 (20.0)	32 (80.0)	

### Knockdown of EMP3 suppresses cell proliferation, cell cycle progression, and tumor growth of HCC

**Figure 2:**
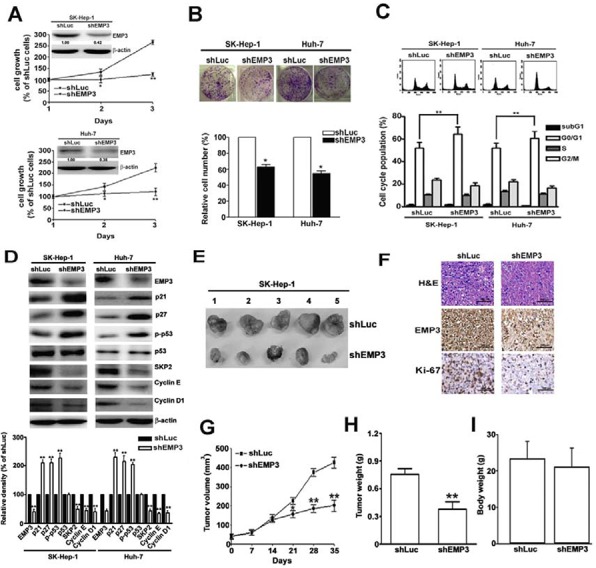
Knockdown of EMP3 suppresses cell proliferation, cell cycle progression, and tumor growth of HCC **A.** Inserted plot: the amounts of EMP3 determined by immunoblotting in the shLu- and shEMP3-SK-Hep-1 and Huh-7 cells. The cell growth curve of each infected cell line was determined by MTT assay. **B.** The clonogenic ability of each infected cell line was examined by colony formation assay. The bottom plot was the quantitative results comparing to that of shLuc-infected cells. **C.** Upper plot: the representative results of cell cycle distribution determined by flow cytometer. The bottom plot was the percentage of cell distribution at sub-G1, G1, S, and G2/M phases of shLuc or shEMP3 cells. **D.** The expression of EMP3 and cell cycle related proteins as indicated were determined by immunoblotting. The relative amount of each protein comparing to that in shLuc cells was shown in the bottom. Nude mice were subcutaneously inoculated with 5 × 10^6^ shLuc- or shEMP3-cells and the tumor volume was measured at indicated time intervals. The representative results of tumor mass in each group were shown in **E.** The representative results of IHC staining against EMP3 and Ki-67 and hematoxylin and eosin (H & E) staining of tissue sections were shown in **F.** Scale bars = 100 μm. **G.** The volume and **H.** weight of tumor mass, and **I.** the body weight from 5 mice were shown, respectively. Data are presented as the mean ± SE of at least three independent experiments. *, *p* < 0.05; **, *p* < 0.01.

To determine the effects of EMP3 on HCC cells, EMP3 was knocked down by the specific short hairpin RNA (shRNA) in SK-Hep-1 and Huh-7 cells. Knockdown of EMP3 markedly suppressed cell proliferation in both cell lines as measured by cell growth (Figure 2A) and clonogenic property (Figure 2B). In addition, knockdown of EMP3 resulted in cell cycle arrest at G1 phase (Figure 2C) and increasing the levels of cyclin-dependent kinase inhibitors, p21, p27, and the phosphorylation status of p53, but reducing the levels of SKP2, cyclin E, and cyclin D1 (Figure 2D). These results suggest that EMP3 is involved in regulation of cell proliferation and cell cycle progression of HCC cells *in vitro*.

To address the role of EMP3 in tumorigenesis of HCC *in vivo*, the EMP3-knockdown (shEMP3)- or Luc-knockdown (shLuc)-SK-Hep-1 cells were injected subcutaneously and the tumor growth was measured (Figure 2E). The expressions of EMP3 and a proliferation marker, Ki-67, were much lower in the tissue sections from shEMP3-SK-Hep-1 cell-inoculated mice than that from control shLuc-SK-Hep-1 cell-inoculated one (Figure 2F). The tumor volume (Figure 2G) and tumor weight (Figure 2H) were significantly decreased in shEMP3-SK-Hep-1 cell -inoculated mice without altering the body weight (Figure 2I). These results provided the evidence that EMP3 is conversely correlated to tumorigenesis of HCC *in vivo*.

### Knockdown of EMP3 inhibits the migratory and invasive abilities of HCC cells through down-regulation of MMP-9 and uPA

**Figure 3:**
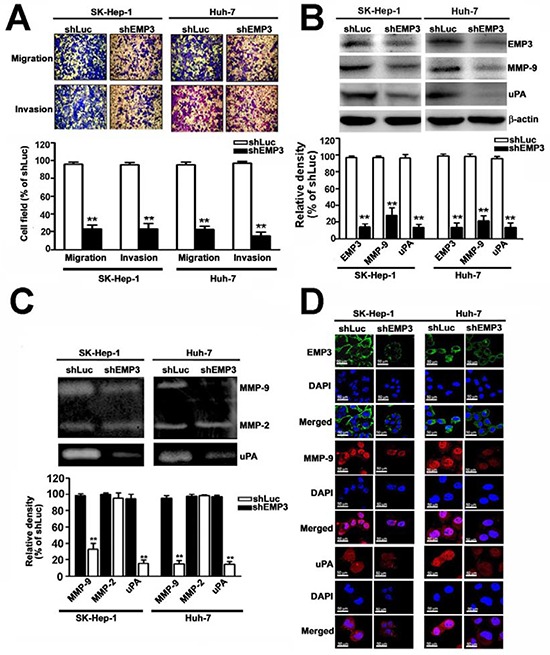
Knockdown of EMP3 inhibits the abilities of migration and invasion of HCC cells through down-regulation of MMP-9 and uPA **A.** Upper plot: the representative results of the *in vitro* migration and invasion assay. Lower plot: the relative abilities of migration and invasion of shEMP3 cells was compared to that of shLuc cells. **B.** The protein expressions of EMP3, MMP-9, and uPA were examined by immunoblotting. β-actin was an internal control. The relative amount of indicated protein was shown in the bottom plot from 3 independent experiments. **C.** Upper panel: the proteolytic activity was examined by zymography. Lower plot: the relative density of proteolytic band of MMP-9, MMP-2, and uPA from 3 independent experiments. **D.** The IF staining of EMP3 (green), MMP-9 (red), and uPA (red) and DAPI staining of nucleus (blue color) in each cell line. Scale bars = 50 μm. Data are presented as the mean ± SE of at least three independent experiments. **, *p* < 0.01.

The effects of EMP3 on malignancy of HCC cells were further examined by *in vitro* cell migration and invasion assays. Knockdown of EMP3 dramatically reduced the migratory and invasive abilities of both SK-Hep-1 and Huh-7 cells (Figure 3A). While the expression of EMP3 was decreased in the shEMP3-cells, the expressions of MMP-9 and uPA were significantly reduced in comparison with that in the control shLuc-cells (Figure 3B). The results from zymography revealed that proteolytic activities of MMP-9 and uPA were obviously reduced after knockdown of EMP3; however, MMP2 activity did not altered (Figure 3C). The reduced levels of MMP-9 and uPA after knockdown of EMP3 were also observed by immunofluorescence staining (Figure 3D). Taken together, the results suggest that silencing EMP3-recuced migratory and invasive abilities of HCC cells might be properly due to suppression of MMP-9 and uPA.

### EMP3-regulated cell proliferation and malignancy of HCC cells are mainly through PI3K/AKT pathway

**Figure 4:**
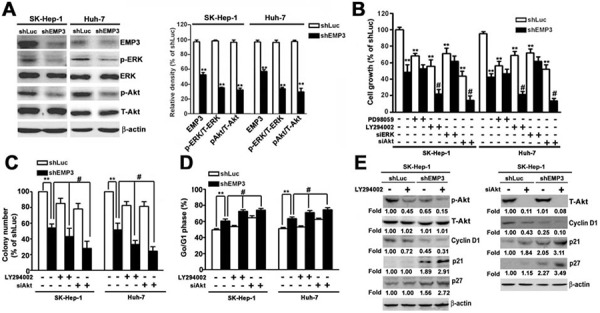
Knockdown of EMP3 suppresses cell proliferation of HCC cells mainly through inactivation of PI3K/Akt pathway **A.** The expressions of indicated proteins were examined by immunoblotting. The relative amounts of indicated proteins in shEMP3 cells comparing to that in shLuc-infected cells were shown in the right plot. **B.** Cells were treated with or without 30 μM of PD98059 (a MEK inhibitor), or 30 μM of LY294002 (a PI3K inhibitor) for 2 h, or transfected with siRNA towards ERK or Akt, and then the cell growth was determined by MTT assay. **C.** Cells were treated with or without 30 μM of LY294002, or transfected with siRNA towards Akt. The clonogenic ability was examined by colony formation assay. The bottom plot was the quantitative results. **D.** Cell cycle was determined by flow cytometer. The percentage of cells distributed at G1 phase. The expressions of indicated proteins were examined by immunoblotting in **E.** Data are presented as the mean ± SE of at least three independent experiments. *, *p* < 0.05, **, *p* < 0.01 compared to the expression levels in shLuc cells; #, *p* < 0.01 compared to the expression levels in untreated shEMP3 cells.

To clarify the signaling pathway of EMP3-regulated cell proliferation and malignancy of HCC cells, the phosphorylation of ERK (p-ERK) and Akt (p-Akt,) were examined. Knockdown of EMP3 significantly reduced the levels of p-ERK and p-Akt, without altering the total amounts of ERK and Akt (Figure 4A). In the control shLuc-cells, disruption of MEK/ERK or PI3K/Akt pathway by a MEK inhibitor, PD98059, or a PI3K inhibitor, LY294002, respectively, suppressed cell growth, and knockdown of ERK or Akt also effectively reduced cell growth (white bars, Figure 4B). In the shEMP3-cells, treatment of LY294002 or knockdown of Akt further enhanced the inhibition of cell growth; however, treatment of PD98059 or knockdown of ERK did not alter shEMP3-reduced cell growth (black bars, Figure 4B). Similarly, disruption of PI3K/Akt pathway by addition of LY294002 or knockdown of Akt also further inhibited the shEMP3-reduced clonogenic ability (black bars, Figure 4C). Moreover, while knockdown of EMP3 resulted in cell cycle arrest at G1 phase in accordance with decreased p-Akt and cyclin D1 and increased p21 and p27, treatment of LY294002 or knockdown of Akt further facilitated EMP3 knockdown-induced G1 arrest (black bars, Figure 4D), p21 and p27 amounts (Figure-4E). Taken together, these results demonstrated that suppression of cell proliferation and induction of cell cycle arrest caused by knockdown of EMP3 were mainly via inactivation of PI3K/Akt pathway.

**Figure 5:**
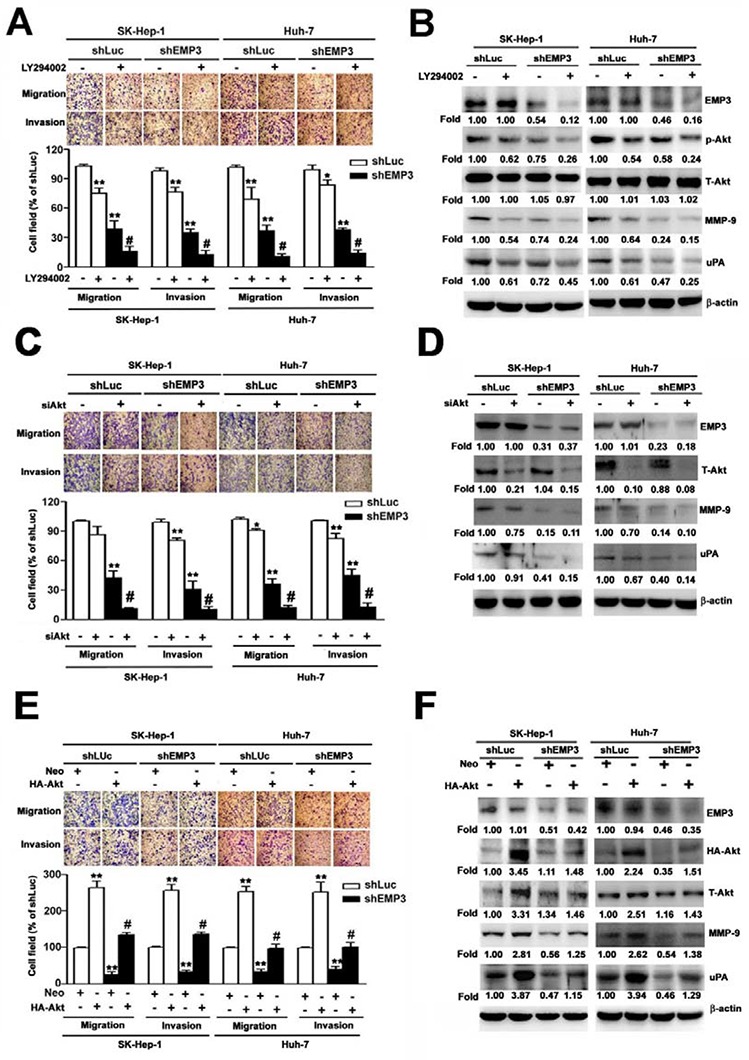
PI3K/Akt pathway regulates MMP-9 and uPA in EMP3-mediated cell migration and invasion of HCC cells **A, B.** Cells were treated with or without 30 μM of LY294002 for 2 h. **C, D.** Cells were silenced by the siRNAs against mock (neo) or Akt (siAkt). **E, F.** Cells were transfected and overexpressed with mock (neo) or HA-tagged Akt (HA-Akt) plasmid. The migratory and invasive abilities were examined in **A, C,** and **E.** The upper plots were the representative results of the *in vitro* cell migration and invasion assay. The relative abilities of migration and invasion were shown in the lower plots. **B, D, F.** The expressions of indicated proteins were determined by immunoblotting. Data are presented as the mean ± SE of at least three independent experiments. **, *p* < 0.01 comparing to that of shLuc cells; #, *p* < 0.01 comparing to that of untreated shEMP3 cells.

While inhibition of PI3K by LY294002 or knockdown of EMP3 suppressed the migratory and invasive abilities (Figure 5A), and reduced the phosphorylation of Akt and the expression of MMP-9 and uPA (Figure 5B) in both SK-Hep-1 and Huh-7 HCC cells, treatment of LY294002 further enhanced the EMP3 knockdown-induced inhibition of the aforementioned phenomena (right black bars, Figure 5A and right lanes, Figure 5B). Moreover, knockdown of Akt synergistically inhibited the EMP3 knockdown-reduced motility and invasiveness (right black bars, Figure 5C), and expressions of MMP-9 and uPA (right lanes, Figure 5D). However, knockdown of ERK did not affect the EMP3 knockdown-reduced malignant properties of migration and invasion in both SK-Hep-1 and Huh-7 HCC cells (Supplemental Figure-1). In contrast, overexpression of Akt increased the migratory and invasive abilities (white bars, Figure 5E) and the expressions of MMP-9 and uPA in the control shLuc- cells (left lanes, Figure 5F). Furthermore, overexpression of Akt rescued the EMP3 knockdown-reduced motility and invasiveness (black bars, Figure 5E) and expressions of MMP-9 and uPA (right lanes, Figure 5F) in the shEMP3-cells. Furthermore, overexpression of EMP3 enhanced malignant properties of Huh-7 cells, including promoting cell growth, migration, and invasion. Identically, overexpression of EMP3 resulted in reduced p21 and p27 levels, increased cyclin D1, uPA, and MMP-9 levels, as well as activation of MMP-9 and uPA activities (Supplemental Figure-2). Taken together, these results indicated that knockdown of EMP3 inhibits the expression of MMP-9 and uPA, and reduces the abilities of migration and invasion is through inactivation of PI3K/Akt pathway. Oppositely, overexpression of EMP3 facilitates aggressiveness of HCC cells.

### EMP3 is clinical relative to the expression of p85, p-AKT, MMP-9, and uPA in the tissue sections from HCC patients

**Figure 6:**
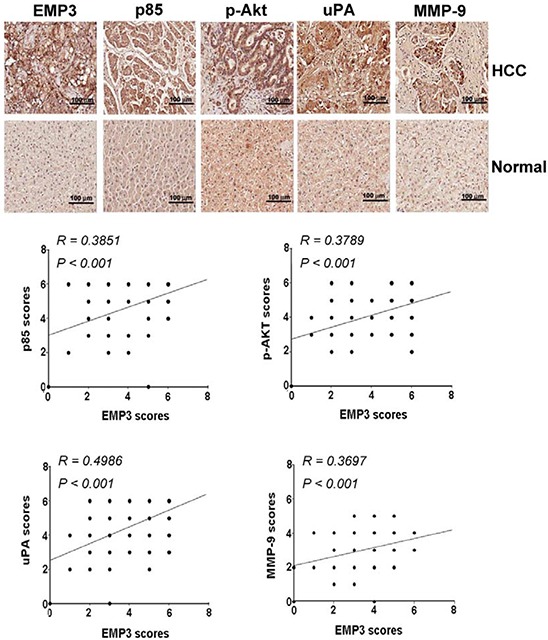
The clinical relevance of EMP3 to p85/p-Akt and uPA/MMP-9 in HCC patients **A.** The indicated protein amounts of the tissue sections from HCC patients were examined by IHC staining and scored from 0 to 8 according to their color density. The upper plots were representative results of IHC staining against indicated proteins. The lower plots were the statistical analysis of the correlation of EMP3 expression with p85, Akt, uPA and MMP-9 expression in HCC tissues. Scale bars = 100 μm.

To determine the potential clinical significance of EMP3 and PI3K/Akt and uPA/MMP-9 pathways, the tissue sections from clinical HCC patients were collected and applied to immunohistochemical (IHC) staining against these proteins. As shown in Figure 6, the expression of EMP3 was significantly positive-correlated to the expression of p85 regulatory subunit of PI3K (*R* =0.3851, *p* < 0.001), p-Akt (*R* = 0.3789, *p* < 0.001), as well as uPA (*R* = 0.4986, *p* < 0.001) and MMP-9 (*R* = 0.3697, *p* < 0.001) in the tissue sections from HCC patients. In accordance with EMP3, the expressions of p85, p-Akt, uPA, and MMP-9 were significantly correlated to poorly differentiated stage in HCC patients. Among these molecules, p85 was also correlated to tumor stage. However, all these molecules were not significantly related to HCC patients’ age and gender (Table 1). Taken together, these results suggest that EMP3 might up-regulate PI3K/Akt pathway and uPA/MMP-9 cascade, leading to progression of HCC development.

### Knockdown of EMP3 reduces the expression and association of p85 regulatory subunit and inhibits PI3K activity

**Figure 7:**
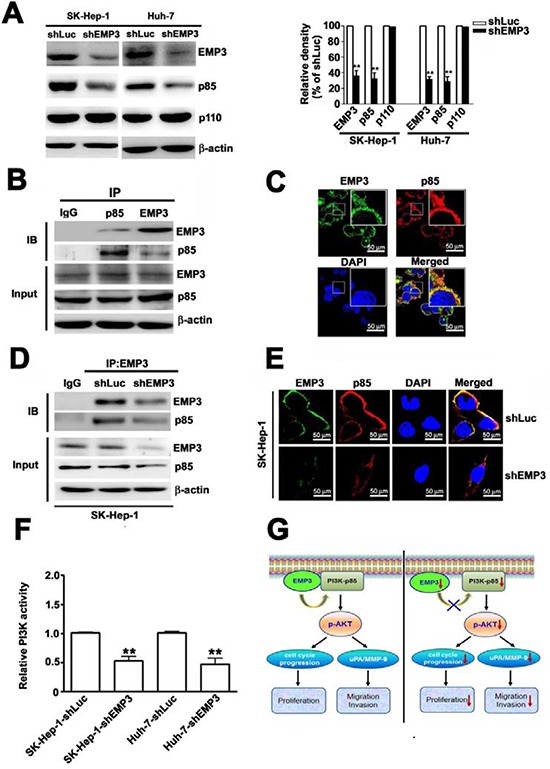
Knockdown of EMP3 reduces the expression and activity of PI3K in SK-Hep-1 cells and the proposed roles of EMP3 in HCC proliferation and invasion **A.** The protein amounts of EMP3, p85 regulatory subunit and p110 catalytic subunit of PI3K, and β-actin were determined by immunoblotting. The quantitative amounts of indicated proteins were shown in the right plot. **B.** The association between EMP3 and p85 in SK-Hep-1 cells was examined by the reciprocal immunoprecipitation (IP) / immunoblotting (IB) with anti-EMP3 and anti-p85 antibodies as indicated in the upper panel. The lower panel showed the input of the indicated protein in cell lysate. **C.** The localization of EMP3 (green) and p85 (red) in SK-Hep-1 cells were determined by IF staining. The cell nucleus was stained with DAPI (blue). **D.** The association of EMP3 and p85 in shLuc-SK-Hep-1 cells and in shEMP3-SK-Hep-1 cells were examined by IP/IB against EMP3 and p85, respectively. The lower panel showed the input of the indicated protein in cell lysate. **E.** The localization of EMP3 (green) and p85 (red) were determined by IF staining and cell nucleus was stained with DAPI (blue) in SK-Hep-1 cells. **F.** The relative kinase activity of PI3K was shown. **G.** The role of EMP3 in cell proliferation and invasion of HCC was illustrated. Left plot: EMP3 associates with PI3K-p85 involved in activation of PI3K/Akt pathway to promote cell cycle progression and proliferation, and also to enhance uPA/MMP-9 required for migration and invasion in HCC cells. Right plot: targeting EMP3 with shRNA to decrease the level of EMP3 results in reduction of PI3K-p85 and inactivation of PI3K/Akt, which contribute to inhibition of cell proliferation and suppression of migration and invasion by down-regulation of uPA/MMP-9 cascade in HCC cells. Data are presented as the mean ± SE of at least three independent experiments. **, *p* < 0.01. Scale bars = 50 μm.

To further explore the role of EMP3 in PI3K/Akt pathway, the expressions of subunits of PI3K and their interaction were determined. Knockdown of EMP3 reduced the expression level of the regulatory subunit of PI3K, p85, but did not alter the expression of the catalytic subunit of PI3K, p110 (Figure 7A). The association between EMP3 and p85 were demonstrated by reciprocal immunoprecipitation (IP)/immunoblotting (IB) approach with anti-EMP3 and anti-p85 antibodies, respectively (Figure 7B). The co-localization of EMP3 and p85 was also observed by IF staining (Figure 7C). The association and co-localization of EMP3 and p85 were reduced after knockdown of EMP3 (Figures 7D and 7E). The results from functional analysis of PI3K revealed that knockdown of EMP3 significantly inhibited the kinase activity of PI3K in both SK-Hep-1 and Huh-7 cells (Figure 7F). These results suggested that knockdown of EMP3 decreases the expression of p85 and inhibits PI3K activity, leading to inactivation of Akt.

In summary, we provided evidence showing that EMP3 associates with PI3K-p85 involved in activation of PI3K/Akt pathway to promote cell cycle progression and proliferation, and also to enhance uPA/MMP-9 required for migration and invasion in HCC cells (left, Figure 7G); in contrast, targeting EMP3 with shRNA to decrease the level of EMP3 results in reduction of PI3K-p85 and inactivation of PI3K/Akt, which contribute to inhibition of cell proliferation by decreased cycle progression at G1 phase, and suppression of migration and invasion by down-regulation of uPA/MMP-9 cascade in HCC cells (right, Figure 7G). The findings suggest that EMP3 might serve as a potential therapeutic target for HCC.

## DISCUSSION

EMP3 shares approximate 40% amino acid identity with PMP22, which is the best characterized member of the family and implicated in the regulation of cell proliferation [11]. Accumulated evidence suggested that EMP3 might be a tumor suppressor gene in glioma [13–16], neuroblastoma [13, 17], esophageal squamous cell carcinoma (ESCC) cell lines [20], and non-small cell lung cancer (NSCLC) [18]. Treating neuroblastoma cell lines with a demethylating agent, 5-aza-2-deoxycytidine, to restore EMP3 expression reduced colony formation density and tumor growth in nude mouse xenograft models [13]. Overexpression of EMP3 resulted in growth inhibition and TERT repression in ESCC cell lines [20]. Releasing A549, a NSCLC cell line, from serum starvation to trigger its cell cycle progression repressed the expression of EMP3 [18]. However, overexpression of EMP3 is related to retain chromosomes 1p and 19q in oligodendroglial tumors [21], and relevant to histological grade III, lymph node metastasis, and strong Her-2 expression in primary breast carcinoma [22]. Recently, functional crosstalk between EMP3 and ErbB2 has been reported. Overexpression of EMP3 promotes cell proliferation and migration and is clinically associated with poor patient survival and metastasis-free survival. Co-expression of EMP3 and ErbB2 is worse for progression-free and metastasis-free survival in patients with upper urinary tract urothelial carcinoma [23]. In our study, we found that knockdown of EMP3 suppresses cell proliferation, cell cycle progression, migration, invasion, and tumor growth of HCC cells. Clinical significance is also observed, namely higher expression of EMP3 in tumorous tissues and associated with poor differentiated stage of HCC patients. These aforementioned studies indicate the potential tumor promoting functions of EMP3 in other cancer types including oligodendroglioma, breast carcinoma, urothelial carcinoma, and hepatoma, suggesting that the roles of EMP3 in cancer progression might depend on cancer types.

The activation of p110 catalytic subunit of PI3K results from the binding of its p85 regulatory subunit to receptor tyrosine kinases (RTKs). Activated PI3K catalyzes the formation of phosphatidylinositol 3,4,5 trisphosphate (PIP_3_), which in turn activates phosphoinositide-dependent kinase-1 (PDK1), leading to activation of Akt. The PI3K/Akt signaling pathway affects multiple cellular processes including cell proliferation, survival, and motility [25]. Activation of PI3K by ErbB2, which has kinase activity and lacks p85-binding sites, depends on assistance of ErbB3, which lacks tyrosine kinase activity and possesses six p85-binding sites, for recruitment of PI3K. Transactivation of heterodimer of ErbB2-ErbB3 is important oncogenic signaling involved in the PI3K/Akt pathway [26]. Recently, it has been reported that ErbB2 and EMP3 reciprocally up-regulate each other accompanied with activation of PI3K/Akt pathway to promote proliferation and migration of human bladder cancer cells [23]. In the present study, knockdown of EMP3 reduces the expression and association of p85 subunit of PI3K to inactivate Akt, leading to inhibition of cell proliferation, migration, and invasion of HCC cells. It is possible that EMP3-regulated p85 expression and association might be probably related to expression or activation of RTKs, such as ErbB2-ErbB3. In addition, it has been reported that EMP3 interacts with C terminus of P2X7 receptor (P2X7R) involved in cell blebbing [27]. Recently, the promoting effects of P2X7R on tumor growth have been demonstrated *in vivo*, and the clinical positive-correlation of P2X7R has been also observed in many cancers [28]. The roles of EMP3 in activation of PI3K by ErbB2-ErbB3 and in tumor promotion by P2X7R need to be further explored.

In addition, EMP-1, one of EMP family members, has also been reported to be involved in activation of PI3K/Akt pathway in lung cancer. They demonstrated that EMP-1 was significantly up-regulated in NSCLC patients comparing to that in benign patients. Overexpression of EMP-1 resulted in increasing cell proliferation of PC9 NSCLC cells in accordance with activation of the PI3K/Akt pathway. EMP-1 promoted tumor growth of PC9 cells was also observed *in vivo* in a nude mouse xenograft model [29]. In contrast, the expression of EMP-3 was suppressed in proliferating A549 NSCLC cells [18]. Although the amino acid sequences of EMP family members are highly homologous, their functions in lung cancer progression are diverse, suggesting that the downstream signaling pathways of EMPs are critical in their effect on tumor progression. Activation of the PI3K/Akt pathway by EMP-1 in NSCLC cells [18] or by EMP-3 in HCC cells in the current study promoted tumor aggressiveness.

The uPA/MMPs cascade has been demonstrated to be involved in malignancy of HCC [8, 9], in particular MMP-2 [30–34] and MMP-9 [34–39]. For instance, MMP-2 and uPA are involved in osteopontin-mediated metastasis [33]. Expression of MMP-2 and vascular endothelial growth factor (VEGF) is closely related to invasion and metastasis [30, 31]. Expression of MMP-2 and cytokeratin 19 are independent risk factors for prediction of lymph node metastasis and survival [32]. Several signaling pathways mediated by different modulators have been known to activate MMP-9 in invasion and metastasis of HCC cells, including focal adhesion kinase (FAK)/extracellular signal-regulated kinase (ERK) axis by epimorphin [36], NF-kappa B by interleukin 23 [37], PTEN/PI3K/AKT pathway by Bmi-1 [34]. In contrast, the expression of MMP-9 is repressed by miR-491 [38] and forkhead box transcription factor A2 (FOXA2) [39]. In addition, membrane type 1-matrix metalloproteinase (MT1-MMP) [40] and MMP10 [41] are also involved in metastasis of HCC cells. MT1-MMP increases invasion and intrahepatic metastasis by degradation of ECM, and enhances cell survival upon challenge of detachment of HCC cells [40]. C-terminal truncated hepatitis B virus x (HBx) protein promotes invasiveness and metastasis of HCC cells by activating MMP10 through C-Jun [41]. Our study reveals that EMP3 contributes to tumor aggressiveness by MMP-9 and uPA, but not MMP2, on the PI3K/AKT pathway in HCC cells.

## MATERIALS AND METHODS

### Antibodies and reagents

The antibodies against EMP3, β-actin, p21, p27, SKP2, cyclin E, cyclin D1, MMP-9, uPA, p-ERK1/2, ERK1/2, p-Akt (Ser473) Akt, p85 subunit of PI3K, p110 subunit of PI3K, Ki-67, siRNAs against mock (neo), Akt (siAkt), and (siERK) were purchased from Santa Cruz Biotechnology (Santa Cruz, CA). Horseradish peroxidase-conjugated anti-mouse and anti-rabbit secondary antibodies were obtained from Promega (Madison, WI). MTT [3-(4,5-dimethylthiazol-2-yl)-2, 5-diphenyltetrazolium bromide], propidium iodide (PI), and DAPI (4′-6-diamidino-2-phenylindole) were purchased from Sigma (St. Louis, MO). PD98059 and LY294002 were bought from Calbiochem (San Diego, CA).

### Tissue samples

Sixteen pairs of HCC samples matched to their corresponding non-tumor tissues were obtained from patients who underwent surgical treatment for HCC at Tungs’ Taichung MetroHarbor Hospital in central Taiwan. Tissues from patients were collected after surgical removal, immediately snapfrozen in liquid nitrogen, and stored at −80°C. The study was performed with the Ethics Committee approval by Institutional Review Board, Tungs’ Taichung MetroHarbor Hospital, Taichung, Taiwan (No: 100036)

### Cell culture

The normal liver THLE-2 cells (ATCC CRL-2706) from the American Type Culture Collection (Rockville, MD) were grown in BEGM kit medium (Lonza/Clonetics Corporation, Walkersville) supplemented with gentamycin/Amphotericin (GA) and epinephrine and to which we add extra 5 ng/ml EGF, 70 ng/ml phosphoethanolamine, 10% fetal bovine serum and 100 mg/ml penicillin-streptomycin. Human HCC cell lines, SK-Hep-1 (ACTT. HTB-52) was obtained from the American Type Culture Collection (Rockville, MD, USA). HA22T/VGH (BCRC. 60168), PLC/PRF/5 (BCRC. 60223), and HepG2 (BCRC. RM60025) cell lines were obtained from the Bioresources Collection and Research Center of Food Industry Research and Development Institute (Hsinchu, Taiwan). Huh-7 cells were a gift from Dr. Hui-Ling Chiou. Cells were maintained in DMEM or MEM medium containing 2 mM glutamine, 100 U/m1 penicillin and 100 μg/ml streptomycin (Sigma), and 10% heat-inactivated fetal bovine serum (FBS; HyClone, Logan, UT) at 37°C in a humidified atmosphere with 5% CO_2_. Cells were passaged every 2 days to obtain an exponential growth.

### Immunoblotting

Equal amounts of protein extracts were separated by sodium dodecyl sulfate polyacrylamide gel electrophoresis (SDS-PAGE) and then transferred onto a polyvinylidene fluoride (PVDF) membrane (Millipore, Belford, MA). The membrane was blocked with 5% skim milk at room temperature for 1 h, and then hybridized with specific primary antibody at 4°C overnight. After washing, the membrane was incubated with HRP-conjugated suitable secondary antibody at room temperature for 2 h. Subsequently, proteins were visualized by addition of enhanced chemiluminescence (ECL) reagent (Millipore, Billerica, USA).

### Immunofluorescence (IF) staining

The day before experiment, cells were seeded on an 8 well Lab-Tek Chambered coverglass (Thermo, Rochester, NY). The slides were washed with phosphate-buffered saline (PBS) and cells were fixed with 4% paraformaldehyde, followed by permeabilization with methanol. After washing with PBS, the slides were blocked with 2% bovine serum albumin (BSA), followed by incubation with primary and secondary antibodies in 5% BSA. Subsequently, the slides were mounted in the mounting solution (Invitrogen) containing DAPI for counterstaining cell nucleus. The cell images were observed and photographed under an immunofluorescence microscopy.

### Tissue array and immunohistochemical (IHC) staining

Human HCC tissue array (LV1002) was purchased from US Biomax, Inc., and analyzed according to the manufacturer's instruction. In Brief, the tissue sections were deparaffinized and rehydrated, followed by quenching endogenous tissue peroxides. The slides of HCC tissue array were incubated with 1% hydrogen peroxide at room temperature for 10 min, and then blocked with 5% BSA for 30 min. The slides were initially hybridized with primary antibody (1:100) at room temperature for 1 h. After washing, the slides were hybridized with HRP-conjugated secondary antibody (1:1000) for 30 min, and then visualized by addition of peroxidase substrate for 1–10 min until the desired stain intensity developed. Subsequently, the cells in the tissue sections were counterstained with hematoxylin. The images were observed and photographed under a microscopy

### Gene knockdown by short hairpin RNA (shRNA)

The shRNA delivered by lentivirus system from National RNAi Core Facility (Academia Sinica, Taiwan) was used to knock down a specific gene according to the manual. Briefly, to generate the lentivirus containing specific shRNA, HEK-293T cells were co-transfected with 2.25 μg of pCMV-ΔR8.91 plasmid with Gag and Pol genes, 0.25 μg of pMD.G plasmid with VSV-G gene, and 2.5 μg of pLKO.1 plasmid with specific shRNA for 16 h and then cultured in culture medium containing 1% BSA for another 24 h. The cultured medium containing lentivirus was collected and stored at −80°C as aliquots for further use. To deliver the specific shRNA construct, approximately 80% confluent cells were infected with the lentivirus bearing specific shRNA in culture medium containing 8 μg/mL polybrene and incubated at 37°C for 24 h. Subsequently, cells were subcultured and selected with 4 μg/mL puromycin. The shRNAs against EMP3 is TRCN0000116049 (shEMP3) corresponding to the sequences, 5′-CGCCTTGATCTATGCCATTCA-3′. The shRNA construct against luciferase (shLuc), TRCN0000072244 referring to the sequence, 5, TRCN0000072244 referring was used as a negative control.

### MTT assay

The cell growth was measured by MTT assay. Cells were seeded at a density of 4 × 10^4^ cells/well in a 24-well plate and cultured for indicated time intervals. At each time interval, the medium was replaced with fresh cell culture medium containing 0.5 mg/mL MTT for 4 h. The number of viable cells was proportional to the amount of formazan, a reduction product of MTT, by dehydrogenases in the mitochondria within live cells. Afterward, the medium was removed and the produced formazan was dissolved in isopropanol and measured at 570 nm by a Multiskan MS ELSA reader (Labsystems, Helsinki, Finland). The relative cell number was normalized by the absorbance from the control cells.

### Colony formation assay

Cells were seeded at a density of 5 × 10^3^ cells/well in a six-well plate and cultured for 14 days. The surviving colonies (>50 cells/colony) were stained with 1% crystal violet and the number of colony was counted. The experiments were performed in triplicate.

### Flow cytometric analysis

Cell cycle was analyzed by flow cytometry. Briefly, 2 × 10^5^ cells were harvested and washed with PBS, and then fixed in 75% alcohol at 4°C for 30 min. After washing, cells were re-suspended in 1 m1 of PBS containing 2 mg/m1 of propidium iodide (PI), 0.5 mg/ml RNase A, and 1% Triton X-100 and incubated at 37°C for 30 min in dark. Samples were then detected for their DNA content using a FACSCalibur flow cytometer and the data were analyzed by Cell Quest software (BD Bioscience, Bedford, MA).

### *In vitro* cell migration and invasion assay

*In vitro* cell migration and invasion assay was performed as described previously [24]. For the migration assay, 5 × 10^4^ cells were re-suspended in serum-free medium and placed in the upper chamber of the well insert with 8 μm pore size polycarbonate membrane filter (Millipore). DMEM containing 20% FBS was placed in the lower chamber. For the invasion assay, the experimental procedures are similar to the migration assay as described above, except the well insert was coated with 10 μl Matrigel (5 mg/mL; BD Biosciences) (50 μg/well). After incubation for 18 h or 24 h at 37°C in the migration or invasion assay, respectively, the cells on the upper surface of the membrane were removed by cotton swab, The migrated or invaded cells on the lower surface of the membrane were fixed with methanol and stained with 0.05% Giemsa, and the cells were counted under a light microscope at 200X magnification. This experiment was performed twice independently. The data are presented as mean ± SD of 5 fields from each well of triplicate samples.

### Zymography

The proteolytic activity of MMP-9, MMP-2, and uPA was examined by casein zymography with gelatin and casein, respectively. Cells were cultured in serum-free media for 48 h, and the conditioned medium was then collected. Equal amounts of concentrated media were separated by SDS-PAGE containing 0.2% gelatin or 0.1% casein (Sigma). After electrophoresis, gels were washed with washing buffer (2.5% Triton X-100 in distilled water) three times at room temperature for 20 min. The gel was equilibrated in the developing buffer by gentle agitation at room temperature for 20 min, and then incubated with fresh developing buffer (50 mM Tris–HCl pH 7.5, 0.15 M NaCl, 10 mM CaCl_2_, and 0.05% NaN_3_) at 37°C for 24 h. Bands corresponding to proteolytic activity of MMP-9, MMP-2, and uPA were visualized by negative staining with 0.3% Coomassie blue in 50% methanol and 10% acetic acid.

### *In vivo* xenograft animal model of HCC

The animal experiments were approved by the Institutional Animal Care and Use Committee of Chung Shan Medical University, and the animals were cared for in accordance with institutional guidelines. Approximate five-week-old male nude mice (BALB/c nu/nu, weighing 16–18 g) were purchased from the National Laboratory Animal Center (Taipei, Taiwan). The stable shLuc- or shEMP3-SK-Hep-1 cells (5 × 10^6^/100 μl) selected with puromycin were washed with PBS and subcutaneously inoculated into the left flank of mice (*n* = 5). Tumor size was measured every 7 days using a digital vernier caliper. Tumor volume was calculated with the formula 1/2 L1 (L2)^2^, where L1 and L2 represent the long and short axes of the tumor, respectively. The mice were sacrificed after 35 days. The primary tumors were excised, weighed, photographed, and sectioned for IHC staining.

### PI3K activity assay

Cell lysate was immunoprecipitated with anti-p85 antibody and Protein A/G Agarose beads (Santa Cruz Biotechnology). The measurement of PI3K activity was analyzed by PI3K kinase ELISA assay (Echelon Biosciences, Salt Lake City, UT) according to the manufacturer's instructions.

### Statistical analysis

The results were presented as mean ± standard error (SE) from three independent experiments. Data were analyzed using Instat software (GraphPad Prism4, San Diego, CA). Student's *t*-test or one-way analysis of variance (ANOVA) with a post-hoc analysis using Tukey's multiple-comparison test was used for obtaining parametric data. *P* < 0.05 or *P* < 0.01 was considered to be statistically significant.

## SUPPLEMENTARY DATA AND FIGURES


