# Supplementary material for: Targeting EMP3 suppresses proliferation and invasion of hepatocellular carcinoma cells through inactivation of PI3K/Akt pathway
Source: Oncotarget. 2015 Oct 12;6(33):34859–74. doi: 10.18632/oncotarget.5414 (PMC4741495; doi:10.18632/oncotarget.5414)
Supplement: Supplementary file 1 [file oncotarget-06-34859-s001.pdf]

SUPPLEMENTARY DATA AND FIGURES

Construction of EMP3 plasmid

The constructions of the human EMP3 expression vectors was synthesis form Integrated DNA Technologies (Coralville, Iowa, USA), it were driven by the pIDTSMART vector. The pIDITSMART-EMP3 were isolated and cloned into the pGFP-N1 vector. pGFP-EMP3 plasmid was then constructed by digesting pIDITSMART-EMP3 with HindIII and BamHI isolating the 504 bp fragment,. The pGFP-EMP3 Sequence fidelity of EMP3 was confirmed using DNA sequence analysis.

Transient transfection

For GFP or GFP-EMP3 plasmid transfections, cells were seeded at ~80% confluency and thereafter transfected for 6 h by replacing the previously described culture medium with serum-free DMEM containing 5 µl Lipofectamine 2000 and 3 µg plasmid DNA. Cells were transfected with plasmids encoding full-length EMP3. At the end of the transfection, the Lipofectamine 2000-containing serum-free DMEM was removed and replaced with normal growth medium. The cultures were then incubated for 48 h.

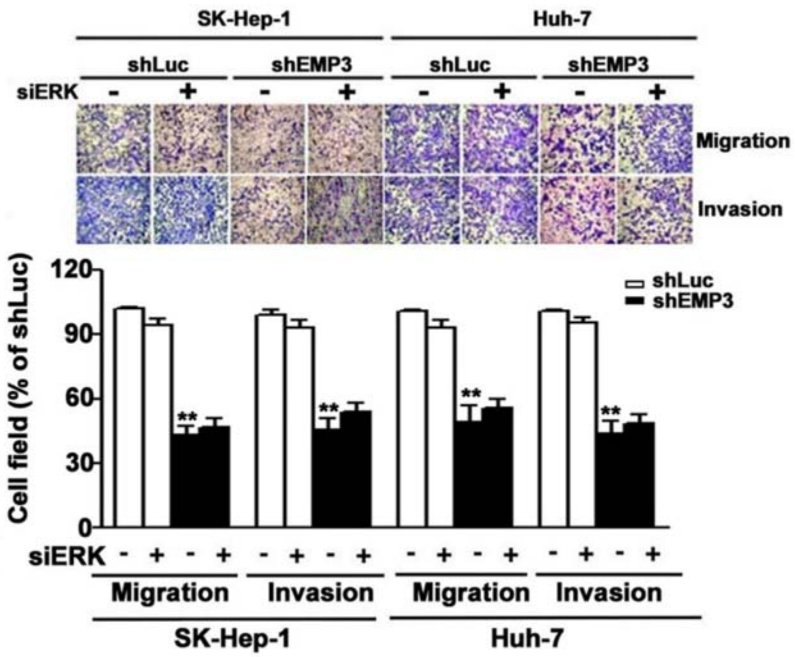

Supplementary Figure S1: Knockdown of ERK does not affect the EMP3 knockdown-reduced migration and invasion in HCC cells. Cells were silenced by the siRNAs against siRNAs against mock (neo-) or ERK (siERK+). The migratory and invasive abilities were examined. The upper plots were the representative results of the *in vitro* cell migration and invasion assay. The relative abilities of migration and invasion were shown in the lower plots. Data are presented as the mean ± SE of at least three independent experiments. **, $p < 0.01$ comparing to that of shLuc cells.

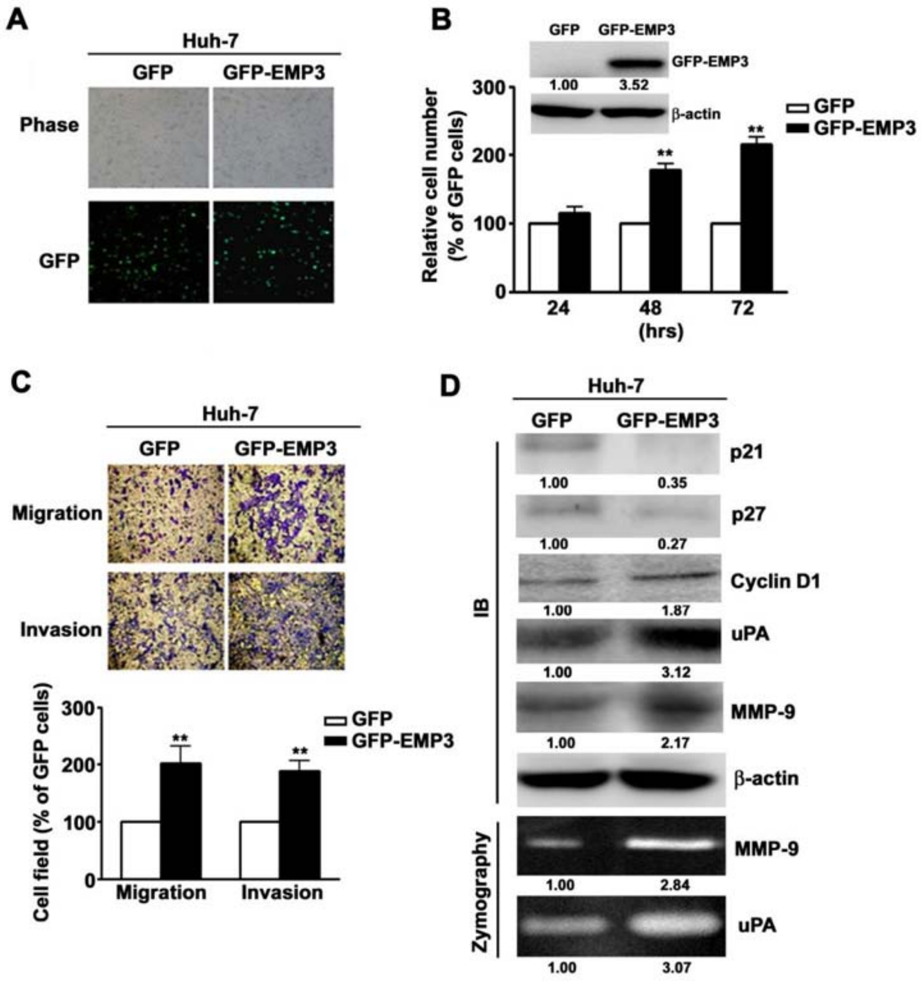

Supplementary Figure S2: Overexpression of EMP3 facilitates aggressiveness of HCC cells. Huh-7 cells were transfected with GFP-plasmid without (GFP) or with EMP3 (GFP-EMP3). **A.** Cell morphology and GFP fluorescence were observed under a microscopy. **B.** The expression of EMP3 was examined by immunoblotting (upper insert). Cell growth was determined by MTT assay. The relative cell number was normalized to that from GFP-transfected cells at 24 h (lower plot). **C.** The representative results of the *in vitro* migration and invasion assay (upper plot). The relative abilities of migration and invasion of GFP-EMP3 transfected cells was compared to that of GFP-transfected cells (lower plot). **D.** The expressions of indicated proteins were determined by immunoblotting (upper plot), and the proteolytic activities of MMP-9 and uPA were examined by zymography (lower plot). Data were presented as the mean ± SE of at least three independent experiments. **, represented $p < 0.01$ comparing to that of GFP-transfected cells.
